# Both the nomogram and the score system can represent an useful tool especially in those cases where the complication is foreseen by the surgeon

**DOI:** 10.1590/S1677-5538.IBJU.2022.0091.1

**Published:** 2022-08-03

**Authors:** Eduardo Mazzucchi

**Affiliations:** 1 Faculdade de Medicina da Universidade de São Paulo - FMUSP Hospital das Clínicas Divisão de Urologia São Paulo SP Brasil Seção de Endourologia, Divisão de Urologia, Hospital das Clínicas, Faculdade de Medicina da Universidade de São Paulo - FMUSP, São Paulo, SP, Brasil

## COMMENT

Urinary Leakage represents in the modern endourology a relatively non-frequent complication after PCNL (3-8.3%). It is generally related to obstructive residual fragments in the ureter, blood clots or simply to pyelo-ureteric junction or ureteral edema. Urinary leakages can be managed conservatively but frequently obligates the surgeon to take the patient back to the operating room to insert a double-J stent or even to remove a ureteral residual fragment (1, 2). Many factors have been implicated in the occurrence of urinary leakage after PCNL including stone burden, high grade hydronephrosis, multiple accesses, a decreased renal parenchyma thickness at the access line, a longer duration of surgery and a prolonged drainage by a nephrostomy tube, among others.

Currently, with the improvement of techniques in PCNL like reducing the caliper of nephroscopes, ultrasound guided punction, flexible nephroscopy at the end of the procedure and tubeless PCNL, the occurrence of leakages tends to reduce. Anyway, to have in mind predictive factors for its occurrence is important once the surgeon can prevent its occurrence inserting a double J stent at the end of the procedure at at particular case for instance. In this retrospective article, the authors identified, in a multivariate analysis, the occurrence of hydronephrosis Grade 2 or more, a renal parenchyma thickness in the access line less than 11.8 mm and the duration of nephroscopy longer than 50 minutes as predictive factors for the development of urinary leakage after PCNL. Based on this they developed a nomogram and proposed an internally validated scoring system with a total score ranging from 3 to 11 where 3-6 represents a low risk, 7-9 moderate risk and 10-11 a high risk for developing urinary leakage after PCNL (3). Both the nomogram and the score system can represent an useful tool especially in those cases where the complication is foreseen by the surgeon. On the other hand, the study was based only in 932 patients (92 patients with urinary leakage and 840 without) studied retrospectively in a single center. Certainly, other series with a bigger number of prospectively studied patients and including other centers is needed for surgeons to adopt it in daily practice.
